# Immune interference in effectiveness of influenza and COVID-19 vaccination

**DOI:** 10.3389/fimmu.2023.1167214

**Published:** 2023-04-19

**Authors:** Yiwen Xie, Xuebin Tian, Xiaodi Zhang, Hangping Yao, Nanping Wu

**Affiliations:** ^1^ State Key Laboratory for Diagnosis and Treatment of Infectious Diseases, The First Affiliated Hospital, Zhejiang University School of Medicine, Hangzhou, Zhejiang, China; ^2^ Jinan Microecological Biomedicine Shandong Laboratory, Jinan, Shandong, China

**Keywords:** immune interference, immune imprinting, antigenic distance hypothesis, influenza vaccine, COVID-19 vaccine, anti-vector immunity, co-administration

## Abstract

Vaccines are known to function as the most effective interventional therapeutics for controlling infectious diseases, including polio, smallpox, rabies, tuberculosis, influenza and SARS-CoV-2. Smallpox has been eliminated completely and polio is almost extinct because of vaccines. Rabies vaccines and Bacille Calmette-Guérin (BCG) vaccines could effectively protect humans against respective infections. However, both influenza vaccines and COVID-19 vaccines are unable to eliminate these two infectious diseases of their highly variable antigenic sites in viral proteins. Vaccine effectiveness (VE) could be negatively influenced (i.e., interfered with) by immune imprinting of previous infections or vaccinations, and repeated vaccinations could interfere with VE against infections due to mismatch between vaccine strains and endemic viral strains. Moreover, VE could also be interfered with when more than one kind of vaccine is administrated concomitantly (i.e., co-administrated), suggesting that the VE could be modulated by the vaccine-induced immunity. In this review, we revisit the evidence that support the interfered VE result from immune imprinting or repeated vaccinations in influenza and COVID-19 vaccine, and the interference in co-administration of these two types of vaccines is also discussed. Regarding the development of next-generation COVID-19 vaccines, the researchers should focus on the induction of cross-reactive T-cell responses and naive B-cell responses to overcome negative effects from the immune system itself. The strategy of co-administrating influenza and COVID-19 vaccine needs to be considered more carefully and more clinical data is needed to verify this strategy to be safe and immunogenic.

## 1 Introduction

As we head towards the third anniversary of COVID-19, more than 761 million people have been infected with COVID-19 and 6.8 million people died worldwide from the SARS-CoV-2 infection (1). The emergence of highly adaptive and more virulent variants of SARS-CoV-2 has significantly increased the COVID-19 mortality rate (2–4). To tackle this alarming situation, multiple vaccines have been developed and approved for emergency use using diverse platforms, for example, mRNA-1273 (Moderna), BNT162b2 (Pfizer/BioNTech), AZD1222 (Oxford/AstraZeneca), and CoronaVac (Sinovac), NVX-CoV2373 (Novavax), Ad5-nCoV (CanSino Biologics/Beijing Institute of Biotechnology) etc. All these vaccines have reportedly exhibited high effectiveness in Phase III clinical trials (5–10). As the ever-evolving seasonal influenza A virus (IAV), antigenic drift caused by mutations of hemagglutinin allows influenza virus to escape the surveillance of immune system, and more importantly, vaccine effectiveness (VE) against IAV has been illustrated to be influenced not only by immune imprinting induced by prior infection or vaccination, but also repeated vaccinations. Highly virulent novel variants of SARS-CoV-2 like Alpha (B.1.1.7), Beta (B.1.351), Gamma (P.1), Delta (B.1.617.2), and Omicron (B.1.1.529) have kept evolving due to continuous genetic mutations in the spike glycoprotein-encoding viral gene (11–13), the VE against SARS-CoV-2 would definitely be influenced like IAV vaccines.

Importantly, co-infection of SARS-CoV-2 with IAV would significantly increase the hospitalization rate, severity rate, and even the mortality rate (14–16). This may result from the unique ability of IAV to raise the expression of ACE2 thus increase the pathogenicity of SARS-CoV-2 (17). Since autumn 2021, the World Health Organization (WHO) has promoted concomitant administration (i.e., co-administration) of inactivated seasonal influenza vaccines along with any kind of COVID-19 vaccines in distinct anatomic sites for the 2021–2022 flu season (18). Following the WHO guidelines, several countries, including Finland, France, Germany, Italy, Spain, Switzerland, and the UK, have also adopted the co-administration strategy for vaccinating their people against SARS-CoV-2 (19–24).

The effectiveness of a vaccine can be regulated by multiple physiological and environmental factors, including sex, age, ethnicity, immunocompromised conditions, and most importantly, the immune system itself. Importantly, previous studies have demonstrated that the effectiveness of the influenza vaccine can be interfered with either by prior vaccination against IAV or by repeat vaccinations (25), and a similar trend has also been observed in the case of COVID-19 vaccination (26). The present literature review is aiming to discuss the negative influence (i.e., interference) on vaccine effectiveness caused by immune imprinting or repeated vaccinations in influenza vaccines and COVID-19 vaccines, and the interference in the co-administration of these two vaccines is also discussed (**Table 1**). Discussions on other non-vaccine-induced factors, such as sex, age, and immunocompromised conditions, are beyond the scope of this review.

**Table 1:** Summary of immune interference in influenza and COVID-19 vaccines.

Mechanisms	Type of vaccine	Impact on immunity or protection	reference
Immune imprinting interferes with immune response against subsequent infections	NM	Increased mortality to subsequent IAV strain	(27, 28)
	NM	Decreased IgG and IgM against SARS-CoV-2 spike and nucleocapsid protein	(29)
	mRNA-1273, mRNA-1273.529/BNT162b2	A sustained hierarchical Spike-binding antibody response to the SARS-CoV-2 variants	(30, 31)
Repeated vaccinations lead to a refractory state of CD4^+^ T-cells and impair antibody production	Fluzone, Flucelvax, Flublok/Vaxigrip Tetra, Influsplit Tetra, Influvac Tetra	Diminished CD4^+^ T-cell responses and attenuated antibody responses	(32, 33)
Pre-existing anti-vector immunity interferes with response to booster dose	ChAdOx1 nCoV-19 vaccine	Impaired VE against SARS-CoV-2 infections	(34, 35)
Short dose interval interferes with maturation of CD4^+^ T-cells	BNT162b2	Improved VE after extending dosing interval	(36–38)
The sticky mucous of respiratory tract interferes with the production of mucosal immunity	AZD1222	Neither mucosal antibodies nor systemic immune response are induced	(39)
Co-administration of influenza and COVID-19 vaccines interferes with immune responses but the underlining mechanism is unknown	NVX-CoV2373+ Flucelvax Quadrivalent/Cominarty + Flucelvax	Impaired immunogenicity of NVX-CoV2373	(40, 41)
	CoronaVac+IIV4 (Sinovac Biotech)	Impaired immunogenicity of CoronaVac and enhanced immunogenicity of IIV4	(42)

NM, not mentioned in original article; IIV4, inactivated quadrivalent inflfluenza vaccine.

## 2 Immune imprinting of IAV interferes with VE against infections

In the late 1950s, Thomas Francis et al. first illustrated that an individual who was first exposed to a specific variant of a virus during his childhood would generate relatively high levels of antibodies against that viral strain, even when exposed to other antigenically distinct IAVs, and was named the original antigenic sin (OAS) theory (43). Plenty of clinical clinical investigations have been conducted in humans (44, 45) and animals (46, 47), finally proving the physiological basis of this theory. During the H1N1 influenza pandemic in 2009, people older than 65 years were better protected and showed a surprisingly lower disease severities compared with younger adults (48). Follow-up examinations then detected that a subset of older individuals (age ≥65 years) already had high levels of preexisting haemagglutination inhibiting (HAI) antibodies cross-reactive to the pandemic H1N1/pdm09 viral strain (49–51), which circulated continually from 1918 to 1957. Hence, most older people born before 1957 had been infected primarily with antigenically similar subtype H1N1 during their childhood, and that might have protected them in the 2009 H1N1 pandemic. Likewise, a Japanese study has reported that individuals previously infected with IAV in the 1918 pandemic or an antigenically similar strain had very high titers of neutralizing antibodies. Importantly, the authors found that the pre-existing immunity could still provide powerful protection even the 2009 H1N1 strain exhibited enhanced pathogenicity in mice- and ferrets-models than other seasonal H1N1 strains (52). Conversely, if the pandemic viral strain encountered during early life is antigenically distinct from the subsequent strains, susceptibility to subsequent strains might increase by several folds. Such phenomena eventually occurred during the 1918 Spanish flu pandemic, where the mortality peaked in individuals of ages around 28 years (27). Using data from Canada and the U.S., the authors speculated that the increased mortality might be attributed to the earlier infection of the Russian flu pandemic strain in 1889–90, as opposed to dominant H1N1 in 1918 (27). A similar incident occurred in the 2009 H1N1 pandemic, where individuals born during the 1957 H2N2 Asian flu pandemic showed enhanced susceptibility to infection and even death (28).

The initial investigation on OAS did not find any interference in immune responses to subsequent vaccinations but rather a strong memory response to the original infecting strain. While emerging studies have shown contradictory findings. In a ferret-model study, seronegative ferrets were sequentially infected with different subtypes of H3N2 viruses showing extended cross-reactivity to all tested influenza clades at the expense of hemagglutination inhibitory (HAI) titers against the current endemic strain (53). Furthermore, a recent study has found that the pre-existing immunity induced by prior infections or vaccinations could modulate distinct immune responses, causing a bias in the antibody-mediated immunity towards conserved yet non-protective IAV epitopes, resulting in minimal immune protection. On the other hand, prior vaccinations can boost the cross-reactivity of neutralizing antibodies against the HA head of IAV, thereby facilitating the body’s immune defense (54). Besides, in a population-based study, the authors combined age and levels of neutralization antibody against the H3N2 strains circulating from 1968 to 2008 into a statistical model. The results showed that HAI titers were the highest against strains infected in the age range of 5–10 years, while antibody titers from subsequent infections gradually dropped with age (55), suggesting a previously unexplored role of OAS in inducing the antibody production against infections and particularly during the vaccination.

Notably, the mechanism underlying the OAS theory remains to be elucidated. There is an opinion regarding the competency of memory versus naive B cells that the memory B cells are higher in proportion and require a lower threshold to be activated compared to those of naïve B cells (56, 57). By tracing the fate of antigen-specific precursors, memory B cells expressing isotype-switched immunoglobulins (swIg) could be activated more promptly than their naive counterparts with the coordination of high-affinity neutralizing serum Ig (58). High-resolution proteomic analysis of Ig and high-throughput transcriptomic analysis of B-cell receptor genes found pre-vaccination antibody titers are positively correlated with the post-vaccination serum B-cell repertoires, as opposed to inducing *de novo* antibody responses (59). Furthermore, a mouse-model study has tried to figure out the extent to which antigen stimulation could induce OAS. Interestingly, OAS was observed in mice that were sequentially immunized with HA-encoding DNA vaccines as well as those sequentially infected with live influenza virus, on the contrary, there was no OAS in mice sequentially vaccinated with formalin-inactivated viruses (60). This finding is consistent with another study where there was no OAS when human subjects were immunized with inactivated IAV vaccine. It is shown that most of the monoclonal antibodies (mAbs) isolated from single antibody-secreting plasma cells had the highest affinity for the current vaccine strain (61). As IAVs are capable of evolving to escape host defensive immunity, this long-lived humoral immunity generated by the first exposure is limited to a given IAV strain and its closely related strains, indicating that OAS may facilitate the immune escape mechanism of IAV variants.

Since the IAV vaccine has been recommended annually for children aged ≥6 months, children are likely to encounter concomitantly multiple IAV strains, as opposed to a single IAV strain infection. It remains to be determined how the influenza vaccine injected during an individual’s childhood can impact the immune system and whether OAS theory applies to the influenza vaccine encountered during an individual’s childhood. It would be crucial to explore whether priming children with influenza vaccines instead of infection with a single viral strain would induce better antibody-mediated immunity against subsequent IAV infections. A comprehensive understanding of the immune mechanisms of OAS can improve the design and vaccination strategy against life-threatening viral infections.

## 3 Repeated influenza vaccinations can interfere with VE against infections

**Figure 1:**
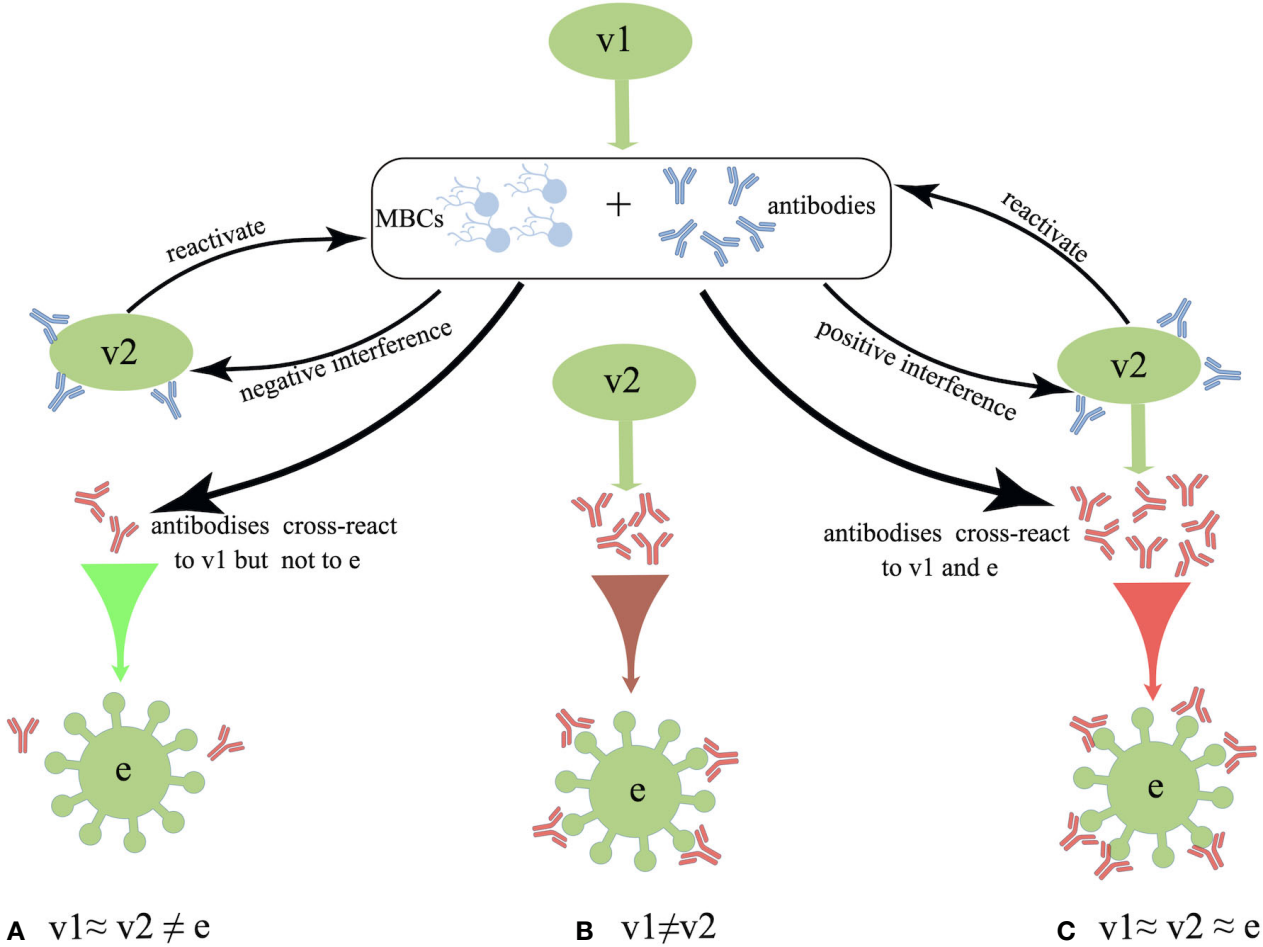
Schematic overview of the antigenic distance hypothesis (ADH). **(A)** Negative interference may occur if the antigenic distance between v1 and v2 is smaller, and that between v1 and endemic strain (e) strains is larger (i.e., v1≈ v2 ≠ e). **(B)** There should be no interference when v2 is antigenically distinct from v1 (i.e., v1 ≠ v2). **(C)** Positive interference may occur when the antigenic distances among v1, v2, and e are relatively small, as these antibodies could react to all three strains (i.e., v1≈ v2 ≈ e).

Interestingly, distinct patterns of B-cell activation and priming are observed between the IAV infection and inactivated influenza vaccination. The repeated inactivated influenza vaccinations resulted in significantly reduced titers of vaccine-specific and cross-reactive antibodies induced from subsequent vaccines, whereas previous exposure to a natural influenza A(H1N1)pdm09 strain did not affect the plasma-blast response to the subsequent vaccines (62). In the 1970s, Hoskins et al. first observed potentially negative effects of repeated vaccinations on pediatric subjects, where repeatedly (2-3 doses) vaccinated children failed to exhibit any superior protection against IAVs compared with the unvaccinated subjects (63–65). Moreover, repeatedly vaccinated children presented approximately 50% higher rate of infections than the immunologically naive children due to an antigenic mismatch between the A/Victoria viral strain circulating during the 1970s and the vaccinated strain (65). Later in 1999, a computer model was used to simulate the immune responses to IAV and predict the vaccine effectiveness. The authors further proposed the antigenic distance hypothesis (ADH) theory, stating that the vaccine efficacy in repeatedly vaccinated individuals can widely vary depending on the antigenic distance among previous vaccine strain (v1), current vaccine strain (v2) and endemic strain (e) based on the corresponding hemagglutination inhibition (HAI) assay (66) (summarized in **Figure 1**).

Since the 2004–2005 season, the Canadian Sentinel Practitioner Surveillance Network (SPSN) has proposed the annual test-negative design (TND), which involves the collection of specimens from patients with influenza-like symptoms and assessment of vaccine effectiveness into a framework (67–76) (shown in **Table 1**), that is conceivable by randomized controlled trials (RCTs) (77, 78). Based on this network, during the 2012-2013 episode, in which the vaccine strain was similar but not homologous to a prior antigenically distinct endemic strain (i.e., v1≈ v2 ≠ e), the VE against the H3N2 strain was found to reduce in repeatedly vaccinated subjects compared with that of the first-time vaccinees (69). Subsequently, during the 2014–2015 influenza season, due to the newly mutated glycosylation site in the circulating H3N2 strain, the endemic strain A(H3N2) subtype presents antigenically distinct variants than v1 and v2 in Canada. Even though v1 and v2 are homologous strains (i.e., v1 = v2 ≠ e), the VE against the H3N2 in repeat vaccinees was historically low (71). Moreover, consistent with the ADH, Canadians who were both vaccinated in 2015-2016 and 2016-2017 seasons were observed to have a preserved VE against infection, as the vaccine strains in these two seasons were antigenically distinct (i.e., v1 ≠ v2) (73). Apart from Canada, a US study also monitored the relative effectiveness of influenza vaccines between the 2004–2005 and 2012–2013 influenza seasons, revealing that the VE against the H3N2 and influenza B strains were both significantly lower among the individuals with a history of repeated vaccinations compared with that in vaccine naïve subjects (79). Despite this, a great negative dose-response pattern for A(H3N2) has been observed in those who were vaccinated in 3 consecutive seasons since the 2012-2013 season. The study showed that the risk of repeated vaccinees to get infected with IAV was 54% higher than those who were not vaccinated in any season (71). Interestingly, an inverse exposure-response association has been noticed between the number of prior influenza vaccinations (up to four) and the HAI response to A(H3N2) in a cohort of 816 healthcare workers. The geometric mean fold-change ratio (GMR) was reportedly increased from 2.3 in those with prior vaccination of up to 4 doses to 6.2 among the healthcare workers without any prior vaccinations (80).

However, the ADH is not amenable to all conditions. The reduced VE observed during the 2015-2016 season in Canada might also have resulted from other incidences since the v1 and v2 strains were identical and antigenically similar to that of the endemic viral strain on antigenic characterization by the HAI assay. Notably, the genetic evolution of a novel S162N mutated strain in the clade 6B.1 was observed during the 2015–2016 season, and this mutation can change the glycosylation status and shield the K163 epitope of clade 6B viruses which circulated during the 2013–2014 season from vaccine-induced antibodies (72). Also, pre-existing low avidity antibodies may constitute the immune complex and activate the receptors of Fcγ, which may inhibit B-cell response to the later immunization (81). Moreover, the immune complex can mediate the complement activation, resulting in the severe disease following influenza infection (82). As mentioned earlier, ADH alone is not enough to explain the lower VE in repeatedly vaccinated subjects, however, it could function as a useful framework. Undoubtedly, further mechanistic investigations are essential to validate this framework. Of note, the ADH framework can only make relative but not absolute predictions about the effectiveness of a vaccine against viral infection.

In this context, a recent study has revealed that high-affinity HAI antibodies in repeated vaccinees are comparatively short-lived in subsequent years, especially after the first-year vaccination (83), although the total antibodies induced by the first vaccination could be maintained over 600 days (84). Combined with a relatively limited capacity of the immune system to sustain plasma cells (85, 86), high-affinity antibodies generated after the first-year vaccination are thought to be derived from terminally plasma cells and most of which would undergo exhaustion and apoptosis. Interestingly, a longitudinal cohort study has found that the number of vaccine-specific plasma-blasts and the binding reactivities of antibodies are reduced after the second dose of the influenza vaccine and remain substantially low over the next doses (87). Disturbances in the antibody affinity maturation, or even diminished B-cell response to annual vaccination, may account for a decreased VE.

The production and maturation of antibodies by plasma-blasts involve a T-cell dependent maturation process, and the T follicular helper (Tfh) cells have been proven to be an indispensable part of this process (88–90). The Tfh cells have been validated to function as an important regulator of vaccine-induced humoral immunity as the magnitude of IgG antibodies is positively correlated with a subset of circulatory antigen-specific Tfh cells, named ICOS1^+^IL-21^+^CD4^+^ T-cell subset, after the vaccination (91). Strikingly, a study conducted on bulk population suggests that the activation of Tfh cells after vaccination is lower in repeat vaccinees compared to the vaccine naïve subjects (32), arguing that deficient Tfh cell responses in repeatedly vaccinated individuals may account for an attenuated antibody response in repeatedly vaccinated individuals. Interestingly, the expression of CD127 at baseline is positively correlated with strong activation of the Tfh cells (33), and promoting the survival and maintenance of memory T cells (92). Following vaccination, CD127 expression is downregulated on activated CD4^+^ T cells and returns to the baseline level at 28 days when the antigen is eliminated (33). The expression of CD127 on circulatory CD4^+^ T cells may lead to a refractory state, thus impairing the activation of Tfh cells and subsequent antibody production in repeated vaccinees, suggesting that the Tfh cells may play critical roles in the post-vaccination antibody response, and the avidity and the quantity of Tfh cells may also be influenced by repeat vaccinations. However, the underlying mechanism impeding activation of the Tfh cells in repeatedly vaccinated individuals remains to be illustrated. We still lack a comprehensive understanding of how both arms of the immune system respond to repeated vaccinations and interactions between the B and T cells. The incorporation of modern genomic, bioinformatic mapping, and antibody landscape approaches has provided insights into the impact of repeated annual vaccinations on the effectiveness of the vaccine against IAV infection to help guide future vaccine policy recommendations.

As for now, an effective influenza vaccine that exhibit enough effectiveness and provide long-lasting protection against antigen-drift influenza virus, and more importantly, raise no safety concerns, is urgently in need. Some promising influenza vaccines like live attenuated influenza vaccines administrated intranasally (FluMist) (93, 94), M2e-based universal influenza vaccines (95, 96) have shown admirable immunogenic and safe profiles, and more efforts is needed for next-generation influenza vaccines to come through the influenza pandemic.

## 4 Immune imprinting predetermines the VE against the SARS-CoV-2

Since the outbreak of COVID-19, vaccines have exhibited their extraordinary abilities in controlling the pandemic. However, the VE against the SARS-CoV-2 infections could be influenced by multiple factors. Notably, the immune response to subsequent booster doses of COVID-19 vaccines could inevitably be influenced by the immune imprinting derived from prior coronavirus infections or COVID-19 vaccination.

In a longitudinal cohort study, antibodies against seasonal coronaviruses were back-boosted when subjects were infected with SARS-CoV-2. These antibodies are more likely to target the conserved epitopes than variable regions of seasonal coronaviruses. Besides, these pre-existing antibodies are found to negatively interfere with subsequent immune responses against the SARS-CoV-2 infections (29). Interestingly, in a macaque-model study, researchers primed macaques with the mRNA-1273 vaccine (encoding the ancestral virus strain) at weeks 0 and 4, and they found a hierarchical Spike-binding antibody response to the SARS-CoV-2 variants at week 6 (ancestral virus strain>Delta > Beta > Omicron). Then 34 weeks after the second dose of the mRNA-1273 vaccine, macaques were homologously boosted with the mRNA-1273 vaccines or heterologously boosted with the mRNA-1273.529 vaccines (encoding the Omicron strain), and exhibited the same hierarchical Spike-binding antibody responses (30). Furthermore, by profiling polyclonal antibody responses following the COVID-19 mRNA (BNT162b2) vaccination, it was found that individuals first encountered either the Alpha or Delta variant would preferentially generate antibodies towards the receptor-binding domain (RBD) of the Alpha and Delta variant, respectively (31). In contrast, individuals who had been previously immunized with vaccines containing ancestral strains of SARS-CoV-2 were expected to generate antibody responses toward the ancestral viral strains and decreased antibody responses to the Alpha or Delta variant (31). Recent studies have illustrated the mechanism of activating the pre-existing immune response to the Omicron infection (97–99). One study performed a detailed analysis of the specificity, function, and genetic features of memory B-cells (MBCs) following the Omicron infection. The authors found that pre-existing MBCs induced by vaccines were primarily reactivated in the acute phase, exhibiting a broader cross-reactivity to the SARS-CoV-2 variants. However, the Omicron-specific antibodies could not be detected, indicating that the booster dose-induced immune responses mainly recalled the MBCs rather than producing Omicron-specific antibodies (98). Detailed analysis of how the immune system responded to the Omicron infection in healthcare workers who received three doses of COVID-19 vaccines with various conditions of immune imprinting revealed that the B and T-cell responses to the Omicron infection were weakened in individuals who were previously infected and then were vaccinated (hybrid immunity), suggesting that the immune imprinting of previous infections with different SARS-CoV-2 variants could interfere with subsequent immune responses and block the recognition of variable regions of the spike protein of SARS-CoV-2 by the immune system (100).

In contrast to the antibody response, activation of the T-cell response has shown substantial cross-reactivity to the SARS-CoV-2 variants (101–104). A population-based study has demonstrated that the majority of T-cell responses to various SARS-CoV-2 variants (including Omicron) could be maintained 6–7 months after vaccination, even if the vaccine strain is based on the ancestral viral strain (101). In another study evaluating the cross-reactivity of vaccine-induced T-cell responses against the Omicron, both CD8^+^ and CD4^+^ T cells exhibited persistent cross-reactivity to Omicron in 9 months post-vaccination (102). Similarly, individuals who previously immunized the Ad26.COV2.S or BNT162b2 vaccines would generate CD8^+^ and CD4^+^ T cells that broadly cross-react with the Omicron variant despite the substantially reduced neutralizing antibody responses (102). Furthermore, a study testing on the hypothesis that whether T-cell responses induced by various platforms of COVID-19 vaccines could still cross-recognize SARS-CoV-2 variants, it was found that activated T cells presented conserved recognition to the SARS-CoV-2 variants regardless of the vaccine-producing platforms, as opposed to attenuated MBC activation and neutralizing antibody production (101).

The capacity of T cells in exhibiting prolonged cross-reactivity has attracted much attention in recent years (105, 106). It is verified that the T cells can target multiple regions in the spike protein of SARS-CoV-2, thus covering a wide spectrum of mutant variants (105). Accordingly, a review summarizing 25 studies concerning the SARS-CoV-2-derived T-cell epitopes has identified certain T-cell receptors that can recognize over 1,400 different SARS-CoV-2 epitopes (382 for CD4^+^ cells and 1,052 for CD8^+^ cells) (106). Another study has also revealed that the CD4^+^ and CD8^+^ T-cell responses against the SARS-CoV-2 are not merely dominated by the spike protein epitopes. The authors have shown that the CD4^+^ T-cell response can also be directed against epitopes of other virulent factors, including nsp3, nsp4, ORF3s, ORF7a, nsp12, and ORF8, while the CD8^+^ T-cells aim at M, nsp6, ORF3a, and N factors of the SARS-CoV-2 (105). Together, these results may partially explain why vaccine-induced T-cell responses could even cross-react with newly emerging variants, despite their reduced neutralizing antibody response capacities.

It is well recognized that next-generation COVID-19 vaccines should stimulate the production of cross-reactive neutralizing antibodies and robustly induce T-cell responses at the same time. Of note, the naive B cells also need to be engaged for their potential to produce antibodies to tackle various SARS-CoV-2 variants. Since the antibody responses may be impaired by immune imprinting of prior exposure to viral pathogens, more experimental data are urgently needed for a better understanding of the immune imprinting mechanism of COVID-19 vaccines to develop more efficient COVID-19 vaccines in the future.

## 5 Immune interference in VE among varied COVID-19 vaccine platforms

### 5.1 Adenoviral vector vaccine

A clinical trial of the ChAdOx1 nCoV-19 vaccine conducted in Brazil, South Africa, and the UK have reported a negative interference in individuals who have been immunized with two doses of the ChAdOx1 nCoV-19 vaccine. The authors found the effectiveness of the vaccine against symptomatic infections in the LD/SD cohort (individuals who received half of the standard dose (LD) as their first dose and a standard dose (SD) as their second dose) was 90.0% (95% CI: 67.4–97.0), higher than that in the SD/SD cohort (individuals who received SD as their first and second dose). Moreover, the vaccine effectiveness against asymptomatic diseases in the LD/SD cohort was also higher than that in the SD/SD cohort [58.9% (95% CI: 1.0–82.9) vs. 3.8% (95% CI: -72.4 - 46.3)] (34). Subsequently, the same group confirmed in another study containing three clinical trials that the vaccine effectiveness was higher in those who received booster doses more than 12 weeks after the first dose [81.3% (95% CI: 60.3–91.2)] than in those who received booster doses less than 6 weeks after the first dose [55.1% (95% CI: 33.0–69.9)] (35). These findings suggest there may be anti-vector antibodies-induced interference in individuals who are homologously boosted, both primed with a high and short dose.

The vector-induced interference has already been observed in polysaccharide–protein conjugated vaccines (CV) where the immune response to the polysaccharide antigen can be interfered with by the pre-existing anti-vector antibodies (107). For example, when human chorionic gonadotrophin (hCG) is conjugated with the tetanus toxoid (TT), a pre-existing TT-induced immunity can suppress the antibody response to the hCG antigen. The scientific community has proposed the carrier-induced-epitopic suppression (CIES) theory to explain such phenomena. The CIES suggests that pre-existing anti-carrier protein immunity may suppress the immune responses against the hapten or polysaccharide-linked antigens (107).

### 5.2 m-RNA vaccines

Similar to adenoviral vector vaccine, relatively longer interval between doses could also enhance immune response to mRNA vaccines. A comparative study of “short”(2- to 5- week) and “long”(6- to 14- week) interval between doses of BNT162b2 vaccines found long interval was followed by more striking neutralizing antibody and B-cell responses. Of note, the authors observed that long-interval recipients could generate stronger IL-2-secreting CD4^+^ T-cell responses with unchanged or even weaker total T-cell responses (36). More importantly, VE of mRNA vaccines has also been reported to be improved by extended dosing interval (37, 38). Currently, little do we know the exact mechanism leading to such interference, there is an opinion that longer dose interval could provide more time for T cells to maturate, which potentially contributes to stronger immune responses following boost doses.

### 5.3 Intranasal vaccine

As SARS-COV-2 invades host via respiratory tract by binding to ACE2 receptors (108), intranasal COVID-19 vaccines have attracted increasing attention and interest for their extraordinary capacity in inducing mucosal immunity against SARS-COV-2. An adenoviral-vector based intranasal vaccine containing spike protein 1, full-length nucleocapsid protein, and truncated polymerase (AZD1222) has compared the immunogenicity of intranasal and intramuscular injection. Intranasal injection induced stronger circulating antibody responses, tissue-resident memory T-cell responses, trained airway macrophages, and more importantly, intranasal injection exhibited adequate protection against variants of SARS-COV-2 in mouse models (109). Interestingly, a live attenuated influenza virus-based intranasal COVID-19 vaccine encoding the RBD of SARS-COV-2 (dNS1-RBD) not only produced rapid, prolonged local immune responses against SARS-COV-2infection, but also an encouraging role in anti-influenza infection. Such cross-reactive protection may come from the innate response in the nasal and tissue resident T cells in the lung (110). Some other types of intranasal COVID-19 vaccines have also shown promising aspects in fighting against the variants of SARS-COV-2, such as subunit vaccines (111), bacterium-vectored (112) and DNA vaccines (113). However, a failure of AZD1222 in inducing neither mucosal antibodies nor systemic immune response happened (39), it is postulated that the sticky mucous of respiratory tract may halt antigen access and immune activation, thus contributing to the poor immunogenicity of intranasal vaccines (114).

As for now, we have to realize that the only vaccination strategy is not enough to prevent the emergence of new SARS-COV-2 variants and to end the global pandemic, although these vaccines have shown pretty satisfactory abilities in reducing the disease severity and limiting the spread out. It is noteworthy that caution should be taken in making up the vaccine strategies in case of the occurrence of more aggressive variants and to prevent undesired adverse events related to the vaccination. To date, heterologous vaccination regimens (e.g., primed with adenoviral vector vaccines and boosted with mRNA vaccines) have been shown non-inferior or even better effective than the homologous vaccination regimens (115–118). There is still the need to keep the focus on such vaccination strategies and figure out whether other kinds of immune imterference would exist or even get amplified by these heterologous prime-boosting strategies.

## 6 Immune interference in the co-administration of COVID-19 and influenza vaccines

Since SARS-CoV-2 and IAVs are both respiratory viruses and share the same mode of transmission, it is inevitable for someone to be co-infected with both viruses and the co-infection would increase the risk of serious illnesses and death (119, 120). Co-administration of an inactivated seasonal influenza vaccine and any dose of a COVID-19 vaccine has been adopted by multiple regions in the world for easing the burden of healthcare workers. A meta-analysis of mass vaccination has confirmed it as a safe and more effective strategy to control the COVID-19 pandemic (121). However, a version of the immune interference opinion still exists and argues that it might be deleterious to human health in the future.

Co-administration of COVID-19 and influenza vaccines has been verified to be non-inferior in terms of immunogenicity and reactogenicity by three independent RCTs (40, 122, 123), which has validated that the co-administration of both vaccines is safe and minimally toxic. Besides, there was no significant negative impact on the immunogenicity of influenza or COVID-19 vaccination on these three RCTs, except a decrease in the efficacy was observed in an RCT conducted in the UK by Toback et al. (40). In which the participants were randomly assigned to receive two doses of the NVX-CoV2373 (Novavax; Gaithersburg, MD, USA) or placebo (normal saline) 21 days apart, and a dose of influenza vaccine was injected concomitantly with the first dose of NVX-CoV2373 or placebo in the opposite deltoid. A decrease in the geometric mean of enzyme-linked immunosorbent assay (ELISA) value was observed in the co-administration cohort compared with the NVX-CoV2373 alone cohort (40) (Shown in **Table 2**). Furthermore, in seropositive participants presented of this trial, co-administration group also showed immune interference of all ages (40), indicating that the impaired immune response to co-administration could be common regardless of the status of the pre-existing immunity. Similarly, two other studies conducted in China and Italy have also reported interference in individuals co-administrated with COVID-19 and influenza vaccines (41, 42) (Shown in **Table 3**). Interestingly, the Chinese study found the interference was more significant when the influenza vaccine is co-administrated with a booster dose of the COVID-19 vaccine (42).

**Table 2:** VE against influenza infection based on Canada’s sentinel surveillance system from 2010 to 2022 (2020-2021 season is not included in this table for its historically low incidence of influenza infection).

Season (reference)	Vaccine strains of TIV	Circulating strain	VE (95% CI)					
			Overall VE	VE against A(H1N1) pdm09	VE against A/H3N2	VE against B/Yamagata		VE against B/Victoria
2010-2011 (67)	A/(H1N1)pdm09/California/7, A/(H3N2)Victoria/210/2009 (NYMC X-187), B/Brisbane/60/2008(Victoria)-like strains	A(H1N1)pdm09, A/H3N2, influenza B	37 (17-52)	59 (14-80)	39 (14-57)	25 (-18-52) against influenza B		
2011-2012 (68)	A/(H1N1)pdm09/California/7, A/(H3N2)Victoria/210/2009 (NYMC X-187), B/Victoria- Brisbane/60/2008	A(H1N1)pdm09, A/H3N2, B/Victoria, B/Yamagata	59 (43-70)	80 (52-92)	51 (10-73)	71 (40-86)		27 (-21-56)
2012-2013 (69)	A/(H3N2)Victoria/361/2011-IVR-165, A/(H1N1)pdm09/California/7/NYMC-X- 179A/X-181, B/Hubei-Wujiagang/158/2009-NYMC-BX-39	A/H3N2, A(H1N1)pdm09, B/Yamagata, B/Victoria	50 (33-63)	50 (16-80)	41 (17-59)	67 (30-85)		75 (29-91)
2013-2014 (70)	A/(H1N1)pdm09(X-179A/X-181A), A/H3N2-X-223A, B/Yamagata-BX-51B	A(H1N1)pdm09, A/H3N2, B/Yamagata (B/Wisconsin, B/Massachusetts)	68 (58-76)	71 (58-80)	-34 (-280-53)	73 (57-84) (72 (53-83) against B/Massachusetts)		NE
2014-2015 (71)	A/(H1N1)pdm09(X-179A/X-181A), A/H3N2-X-223A, B/Yamagata-BX-51B	A/H3N2, B/Yamagata (B/Wisconsin)	9 (-14-27)	NE	-17 (-50-9)	42 (10-62) (42 (8-63) against B/Wisconsin)		NE
2015-2016 (72)	A/(H1N1)pdm09(X-179A/X-181A), A/H3N2-NIB-88, B/Yamagata-Phuket/3073/2013	A(H1N1)pdm09, A/H3N2, B/Yamagata, B/Victoria	46 (32-57)	43 (25-57)	NE	NE		54 (32-68)
2016-2017 (73)	A/(H1N1)pdm09- California/7, A/H3N2-Hong Kong/4801/2014, B/Victoria- Brisbane/60/2008	A/H3N2, A(H1N1)pdm09, B/Yamagata, B/Victoria	43 (28-55)	NE	36 (18-50)	72 (47-85)		NE
2017-2018 (73)	A/(H1N1)pdm09- Michigan/45/2015, A/H3N2-Hong Kong/4801/2014, B/Victoria- Brisbane/60/2008	A/H3N2, A(H1N1)pdm09, B/Yamagata, B/Victoria	34 (22-45)	52 (18-72)	14 (-8-31)	44 (30-55)		NE
2018-2019 (74)	A/(H1N1)pdm09- Michigan/45/2015, A/H3N2-Hong Kong/4801/2014, B/Victoria- Brisbane/60/2008	A(H1N1)pdm09, A/H3N2, B/Victoria	68 (55-77)	72 (60-81)	NE	NE		NE
2019-2020 (75)	A(H1N1)pdm09 - Brisbane/02/2018, A(H3N2) - Kansas/14/2017, B/Victoria- Brisbane/60/2008	A(H1N1)pdm09, A/H3N2, B/Victoria	58 (47-66)	44 (26-58)	62 (37-77)	69 (57-77) against influenza B		
2021-2022 (76)	A(H1N1)pdm09 - Brisbane/02/2018, A(H3N2) - Kansas/14/2017, B/Victoria- Brisbane/60/2008	A/H3N2	36 (-38-71)	NE	NE	NE	NE	

TIV, trivalent influenza vaccine; CI, confidence interval; VE, vaccine effectiveness; NE, not estimated owing to insufficient sample size.

**Table 3:** Immune interference in the co-administration of COVID-19 and influenza vaccines.

Reference	Vaccination strategy	Immunogenicity assessment	Immunogenicity of COVID-19 vaccine	
			Co-administration group	COVID-19 vaccine alone group
Toback et al. (40)	C+I on day 0, C on day 21 vs. P +I on day 0, P on day 21	ELISA units/ml of anti-S IgG	31236.1 (95% CI: 26295.5-37104.9)	44678.3 (95% CI: 40352.2 -49468.2)
Wang et al. (42)	I + C1 or C1 on day 0, C2 or I + C2 on day 28 vs. C1 on day 0, I on day 14 and C2 on day 28	GMT of neutralizing antibody	27.5 (95% CI: 24.4-31.1)	38.1 (95% CI: 33.6-43.2)
Stefanizzi et al. (41)	I + C3 vs. C3	GMT of anti-S IgG	12343.2 (95% CI: 7,137.7 -26,736.6)	15787.7 (95% CI: 5,829.7 –42,755.8)

P, placebo; C, COVID-19 vaccine; C1, first dose of COVID-19 vaccine; C2, second dose of COVID-19 vaccine; C3, third dose of COVID-19 vaccine I, influenza vaccine; ELISA, enzyme linked immunosorbent assay; GMT, geometric mean titer.

Notably, the decreased immune response of co-administration in all relative studies could still protect participants against infections from the SARS-CoV-2 and IAV variants, and we still lack experimental studies to confirm this interference. Interestingly, the Toback study immunized participants with influenza vaccine concomitantly with the first dose of the COVID-19 vaccine and found a decrease in immune responses against the NVX-CoV2373 vaccine. While the Chinese study reported decreased vaccine effectiveness in participants receiving seasonal influenza vaccine with the booster dose of the COVID-19 vaccine instead of the first dose. This indicates that it is not the immunological memory that modulates such decrease but some other underlying mechanisms waiting to be unraveled. The immunogenicity of the influenza vaccine has reportedly been preserved with concomitant administration in all relative studies (40, 42, 122–124). Surprisingly, the immune response to specific influenza antigen was even stronger in participants co-administrated COVID-19 vaccine and influenza vaccine (42, 122). Compared with influenza vaccine alone group, GMTs against A/H1N1, B/Victoria, and B/Yamagata were higher when BNT162b2 and recombinant quadrivalent influenza vaccine were immunized at the same time (122). Similarly, Stronger immune response against A/H1N1 was also observed in co-administration group when CoronaVac and inactivated quadrivalent influenza vaccine were injected concomitantly (42). These findings suggest something in COVID-19 vaccines may function as an adjuvant to enhance the immunogenicity of influenza vaccine. Of note, it was found the SARS-CoV-2 and most IAVs circulated around the world presently share the same small NGVEGF/NGVKGF peptide. More importantly, this peptide is located in N481-F486 of the RBD of the SARS-CoV-2 and the immunodominant region of the neuraminidase of IAVs, and the pre-existing influenza antibodies could cross-react with the SARS-CoV-2 (125). Whether the small peptide has contributed to immune interference in the co-administration of influenza and COVID-19 vaccine needs further investigation, as the cross-reactive antibodies are non-neutralizing (126).

An alarm has been sounded for us, and we need to bear in mind that there may be an existing immune interference when more doses and a variety of COVID-19 and influenza vaccines are administered concomitantly. Since the heterologous boosting strategy of COVID-19 vaccines has been adopted by several countries, more efforts should be focused on delineating whether a heterologous booster strategy of COVID-19 vaccines with co-administration of influenza vaccines would amplify the interference in the vaccine effectiveness against the infection of both viruses. A better understanding of the impaired immune responses against the SARS-CoV-2 requires advanced genomic tools, proteomics, bioinformatics, and high-throughput immunological assays to reveal the immune interactions between the COVID-19 and influenza vaccines.

## 7 Conclusion

This review aimed to summarize the immune interference in influenza and the COVID-19 vaccines’ effectiveness and how the co-administration of these two types of vaccines would interfere with the activation of the immune system against respiratory virus infections. Distinct from the vaccine failure when Haemophilus influenzae type b (Hib) conjugate vaccine co-administrated with other vaccines (127), most relevant studies have reported an adequate antibody level to protect individuals from infection with the existence of immune interference (40, 42). Of note, we still need to investigate if the antigenic drift of both viruses would cause harm to humans when yearly vaccination schedules of both vaccines are adopted.

Co-administration of influenza and COVID-19 vaccines has already been promoted to ease the burden of public health and even improve compliance with vaccination programs. However, the interactions between these two vaccines have not been unraveled and joint efforts of the scientific community are urgently needed. Interestingly, a combined mRNA vaccine containing both the HA antigen of IAV and the RBD of the SARS-CoV-2 S protein has shown pretty good immunogenicity and safety (128), which provides us with a new approach to develop vaccines against life-threatening infections. Notably, the policymakers need to consider carefully whether or not to adopt the co-administration of these two vaccines nationwide for its uncertainty, and more detailed real-world data plus animal studies are urgently required to facilitate more scientific and effective decision-making.

## Author contributions

YX and XT wrote the manuscript, XZ generated the figures. NW and HY provided critical feedback on the manuscript. All authors contributed to the article and approved the submitted version.
